# Methylenetetrahydrofolate Reductase and Methionine Synthase Reductase Polymorphisms: Genetic Predictors of Congenital Heart Disease Subtypes

**DOI:** 10.7759/cureus.96291

**Published:** 2025-11-07

**Authors:** Sumedha Tripathi, Shally Awasthi, Shalini Tripathi, Amita Jain, Akhil K Sharma

**Affiliations:** 1 Pediatrics, King George's Medical University, Lucknow, IND; 2 Clinical Microbiology, King George's Medical University, Lucknow, IND; 3 Cardiology, King George's Medical University, Lucknow, IND

**Keywords:** atrial septal defect, congenital heart disease, methionine synthase reductase, methylenetetrahydrofolatereductase, patent ductus arteriosus, polymerase chain reaction, restriction fragment length polymorphism, ventricular septal defect

## Abstract

Objectives: The objective of this study was to investigate the association of methylenetetrahydrofolate reductase (*MTHFR*c.677C>T)and methionine synthase reductase (*MTRR *c.66A>G*) *genetic polymorphisms with the occurrence of congenital heart disease (CHD) and its subtypes in children.

Materials and methods: This was a hospital-based case-control which included patients aged 0-11 months hospitalized between July 2022 and December 2023 with an echocardiographically confirmed CHD, and healthy age-matched controls recruited from the immunization clinic, after informed parental consent. Genotyping for *MTHFR*c.677C>Tand *MTRR *c.66A>Gwas performed using polymerase chain reaction-restriction fragment length polymorphism (PCR-RFLP).

Results: A total of 245 cases (73 (29.8%) female infants) and 245 controls (77 (31.4%) female infants) were included. *MTHFR*c.677C>T(TT genotype, OR = 2.75, 95%CI: 1.6-4.5) and *MTRR *c.66A>G (GG genotype, OR = 2.62, 95%CI: 1.6-4.0) were associated with CHD. Subtype analysis showed a higher risk for ventricular septal defect (VSD) (OR = 5.67, 95%CI: 3.1-10.0 ), atrial septal defect (ASD) (OR = 9.51, 95%CI: 4.9-18.4), and patent ductus arteriosus (PDA) (OR=15.52, 95%CI: 7.1-33.7) for *MTHFR *c.677C>T, while *MTRR *c.66A>Gwas associated with ASD (OR = 8.67, 95%CI: 4.9-15.0) and PDA (OR = 18.54, 95%CI:9.3-36.9). The T allele of *MTHFR *c.677C>T and the G allele of *MTRR *c.66A>Gwere significantly more common in CHD cases (OR= 1.73, 95%CI: 1.3-2.2 and OR= 1.85, 95%CI: 1.4-2.3, respectively) compared to controls.

Conclusion: *MTHFR*c.677C>Tand *MTRR *c.66A>Gpolymorphisms are associated with increased CHD risk, particularly in VSD, ASD, and PDA. The higher prevalence of T and G alleles in CHD cases suggests their potential role as genetic risk factors, emphasizing their relevance in CHD susceptibility and risk assessment.

## Introduction

Congenital heart disease (CHD) is among the most common cardiac disorders in children. According to the Global Burden of Disease Study 2017, CHD accounted for nearly 261,000 deaths globally and remains a leading cause of infant morbidity and mortality [1]. In India, CHD contributes significantly to neonatal and infant mortality, with an estimated prevalence similar to global rates but a relatively higher burden due to delayed diagnosis and limited access to paediatric cardiac care [2]. CHD comprises structural and functional cardiac abnormalities present at birth, involving defects in the heart’s walls, valves, or proximal vasculature, which can impair normal hemodynamics. CHDs encompass a wide range of malformations, including obstructive defects, septal defects, cyanotic defects, and hypoplastic anomalies.

CHD is a multifactorial disease with both environmental and genetic factors contributing to its pathogenesis [3]. Environmental contributors during pregnancy include rubella infection in the first trimester, smoking, alcohol consumption, pregestational diabetes, advanced maternal age, obesity, and deficiencies in macro and micro nutrients [4]. Genetic factors include chromosomal abnormalities (e.g., trisomy 13, 18, 21; monosomy X; 22q11.2 deletion), syndromic mutations (*TBX5, JAG1, NOTCH2, RAF1, TFAP2b*), and non-syndromic mutations (*GATA4, NKX2.5, NOTCH1, MYH6, CRELD1*). Additionally, unique copy number variations (CNVs) and single-nucleotide polymorphisms (SNPs), such as *MTHFR* (C677T, A1298C), *VEGF* (C2578A, G1154A, C634G), and *MTRR* (A66G, C524T), are associated with CHD [5,6].

Methylenetetrahydrofolate reductase (*MTHFR*) and methionine synthase reductase (*MTRR*) gene polymorphisms have been implicated in the etiology of CHD and its associated mortality through their influence on homocysteine metabolism. Variations in these genes, such as the *MTHFR* c.677C>T and *MTRR* c.66A>G polymorphism, are associated with impaired enzymatic activity, leading to hyperhomocysteinemia (HHcy) [7,8].

The study aimed to elucidate the association between *MTHFR* c.677C>T and *MTRR* c.66A>G gene polymorphisms and the susceptibility to CHD through a case-control approach. Additionally, the study seeks to evaluate the impact of these polymorphisms on different CHD subtypes.

## Materials and methods

This was a hospital-based case-control study conducted in the Pediatrics department of King George’s Medical University, Lucknow, India, from July 2022 to December 2023. The research protocol was approved by the institutional ethics committee of King George’s Medical University, Lucknow, Uttar Pradesh, India (approval letter no. 115th ECM II B-Ph.D./P2, dated June 21, 2022). Written informed consent was obtained from the legal guardians of all participants, both cases and controls, before recruitment.

Study population

**Figure 1:**
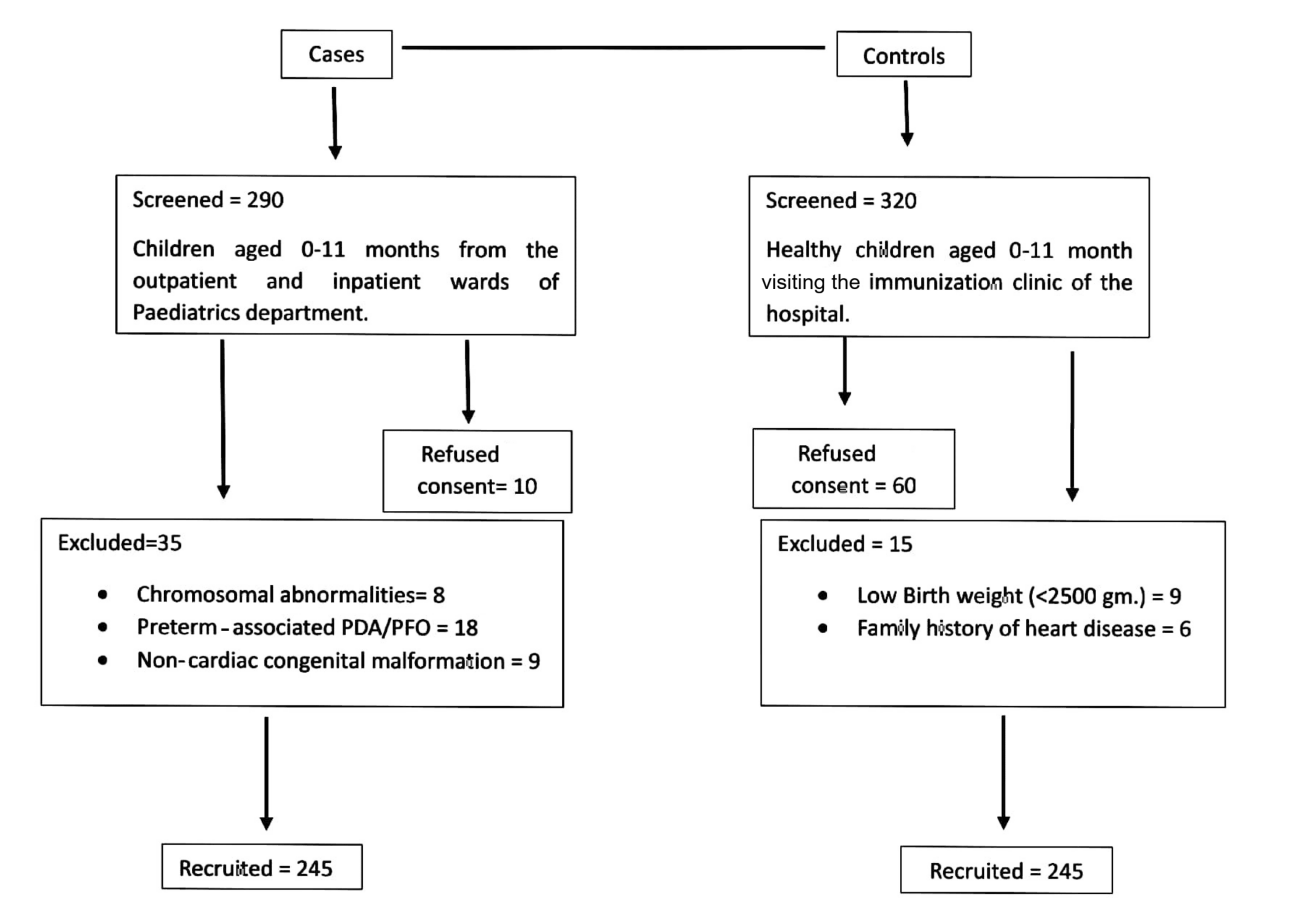
Flow chart of the study. PDA: patent ductus arteriousus; PFO: patent foramen ovale

Cases were recruited from the pediatric inpatient wards, while healthy controls were enrolled from the hospital’s immunization clinic. Figure 1 presents the study design.

Inclusion and Exclusion Criteria of Cases

Included were consecutive patients, aged 0-11 months, hospitalized with clinically suspected and echocardiographically confirmed CHD. Those with clinically diagnosed chromosomal abnormalities, preterm-associated patent ductus arteriosus (PDA) or patent foramen ovale (PFO), or any non-cardiac congenital malformations were excluded.

Inclusion and Exclusion Criteria of Controls

Age-and-gender-matched healthy controls were recruited from the immunization clinic of the pediatric department. Controls with a birth weight below 2500 grams or a family history of heart disease were excluded.

Sample size calculation

Our hypothesis was that the *MTHFR *c.677C>T gene polymorphism was associated with CHD. For cases, the minor allele frequency of the T allele of the *MTHFR* c.677C>T gene polymorphism was assumed to be 0.09 (9%), while for controls, it was taken as 0.03 (3%) based on a previous study conducted in an adult South Indian population [9]. Assuming a statistical power of 80%, a significance level (α) of 0.05 (5%), and a case-to-control ratio of 1:1, a total of 245 cases and 245 controls were required. Online Sample Size Estimator (OSSE) software (https://bio.tools/osse) was used.

Data collection

Data were collected using a pre-designed questionnaire. Parents were interviewed to gather demographic information about the case or control. The socioeconomic status (SES) was evaluated using the Revised Kuppuswamy’s Scale, which categorizes SES into five groups: Lower class, Lower middle class, Upper lower class, Upper middle class, and Upper class. The assessment was based on three parameters: the educational qualification, the occupational status of the head of the household, and the total household income [10]. Clinical findings of investigations, including echocardiography, were extracted from the medical records of the recruited cases. All echocardiograms were done by a single trained cardiologist on the same machine (Vivid E-95; GE Healthcare Technologies, Inc., Chicago, Illinois, United States). Anthropometric measurements, including weight (in kilograms) using an electronic weighing scale, height (in centimeters), and head circumference (in centimeters), were also measured at the time of recruitment. Immunization history was documented based on the information provided in the immunization card.

Sample collection

Under aseptic conditions, whole blood was collected from each participant. Two milliliters of venous blood were drawn in an ethylenediaminetetraacetic acid (EDTA) vial for genetic analysis.

Molecular work

Genomic DNA was extracted from an EDTA whole blood sample using a spin column method according to the protocol (QIAmp, Blood Kit; Qiagen GmbH, Hilden, Germany). DNA was stored at -80°C till the time of use. Purity and quantity were checked by a spectrophotometer at 260/280 nm.

Amplification of *the MTHFR* gene C677T polymorphism

The *MTHFR* c.677C>T polymorphism was genotyped by polymerase chain reaction-restriction fragment length polymorphism (PCR-RFLP). PCR amplification was performed using previously reported primer sequences [11]. The PCR conditions were as follows: initial denaturation at 94°C for five minutes, followed by 40 cycles at 94°C for 30 seconds, annealing at 62°C for 45 seconds and extension at 72°C for 45 seconds. A final extension step was carried out at 72°C for five minutes. RFLP analysis was done by using the Hinf1 restriction enzyme at 37°C for one hour. After restriction fragment length polymorphism (RFLP) analysis, PCR products were separated on 3% agarose stained with ethidium bromide, then visualized by ultraviolet light. Three genotypes were detected: TT (175, 23 bp), CT (198, 175, 23 bp), and CC (198 bp). The 23 bp fragment was not retained on the gel.

Amplification of the *MTRR* gene A66G polymorphism

The *MTRR* C.66A>G polymorphism was genotyped by PCR-RFLP. PCR amplification was performed using previously reported primer sequences [12]. The PCR conditions were as follows: initial denaturation at 95°C for five minutes, followed by 35 cycles at 95°C for 30 seconds, annealing at 59°C for 30 seconds and extension at 72°C for 30 seconds. A final extension step was carried out at 72°C for 10 minutes. RFLP analysis was done by using Nde1 restriction enzyme at 37°C for one hour. After RFLP analysis, the PCR product was separated on 3% agarose stained with ethidium bromide, then visualized by ultraviolet light. Three genotypes were detected: AA (126, 25 bp), AG (151, 126, 25 bp), and GG (151 bp). 

Statistical analysis

Data, entered in MS Excel (Microsoft Corporation, Redmond, Washington, United States) and analyzed using SPSS Statistics for Windows, version 15 (SPSS Inc., Chicago, Illinois, United States) and GraphPad InStat 3 (Dotmatics, Boston, Massachusetts, United States). All the demographic variables were recorded as frequencies and percentages. Chi-square test without Yates correction was used for the categorical variables. Continuous variables were represented as mean ± standard deviation (SD) and analyzed using an independent t-test. For the purpose of analysis, we have dichotomized the variable SES into “Upper or upper middle or upper lower class” and "lower or lower middle class”. The Hardy-Weinberg equilibrium principle was applied to all the cases and controls separately. To better define the risk of various genotypes with CHD, three genetic models (dominant, recessive, and allelic) were used. Risk of CHD or subtypes of CHD was assessed by calculating the odds ratio (OR) with its 95% confidence interval (CI). A two-sided p-value <0.05 was taken as significant.

## Results

A total of 245 cases and 245 healthy controls were included in the study. Among the CHD cases, 73 (29.8%) were female, while among the controls, 77 (31.4%) were female. Table 1 presents a comparison of the sociodemographic characteristics and maternal risk factors between CHD patients and healthy controls. Among case mothers, 65 (26.55%) were aged 30 years or older, compared to 24 (9.8%) in the control group, and this difference was statistically significant (p < 0.001). Additionally, 70 (28.6%) case mothers reported passive smoking exposure during pregnancy, compared to 29 (11.8%) in the control group.

**Table 1:** Sociodemographic characteristics of children with congenital heart disease and healthy controls *p-value was calculated by using the Chi-square test, #p-value was calculated by using the Students’ test SD: standard deviation; BCG: Bacille Calmette–Guerin; DPT: Diphtheria-Pertussis-Tetanus

Characteristics	Cases (n=245)	Controls (n=245)	t- value	χ^2^-value	p-value
*Gender (Female), n (%),	73 (29.8)	77 (31.4)	-	0.15	0.70
^#^Age (in months), n (%)					
< 1	30 (12.2)	41 (16.7)	-	5.06	0.08
1-5	131 (53.5)	107 (43.7)			
5-11	84 (34.3)	97 (39.6)			
^#^Birth weight, (kgs) n (%)					
≤2.5	160 (65.3)	120 (49.0)		12.7	<0.001
>2.5	85 (34.7)	125 (51.0)			
^#^Weight (kgs), mean ± SD	4.50 ± 1.80	5.36 ± 3.54	-3.41	-	0.001
^#^Length (cm), mean ± SD	58.28 ± 8.92	60.02 ± 8.53	-2.21	-	0.03
^#^Head circumference (cm), mean ± SD	39.02 ± 4.69	38.54 ± 4.40	1.16	-	0.25
*Family Type (nuclear), n (%)	30 (12.2)	14 (5.7)	-	6.39	0.01
*Residence (urban), n (%)	60 (24.5)	83 (33.9)	-	5.22	0.02
Immunization status					
*BCG	218 (88.97)	238 (97.14)	-	12.64	0.001
*DPT	65 (26.53)	194 (79.18)	-	136.29	<0.001
*Measles	2 (0.81)	35 (14.28)	-	31.84	<0.001
*Rubella	1 (0.40)	30 (12.24)	-	28.96	<0.001
*Gestational Age, n (%)					
Pre-term	10 (4.1)	9 (3.7)	-	0.06	0.82
*Socioeconomic class, n (%)					
Upper or Upper middle or Upper lower class	125 (51.0)	158 (64.5)	-	9.11	0.003
Lower or lower-middle class	120 (49.0)	87 (35.5)			
^*^Maternal risk factors					
Maternal age (in years), n (%)			-	23.08	<0.001
< 30	180 (73.5)	221 (90.2)			
≥ 30	65 (26.5)	24 (9.8)			
Passive smoking, n (%)	70 (28.6)	29 (11.8)	-	21.28	<0.001
Febrile illness during pregnancy, n (%)	29 (11.8)	18 (7.3)	-	2.85	0.09

The genotype of *MTHFR* c.677C>T (CC, CT, and TT) in the CHD group was 50 (20.4%), 107 (43.6%), and 88 (35.9%), respectively, whereas in the control group, the corresponding frequencies were 80 (32.6%), 114 (46.5%), and 51 (20.8%), respectively. Similarly, for the *MTRR* c.66A>G polymorphism, the genotype distribution of AA, AG, and GG in the CHD was 85 (34.6%), 50 (20.4%), and 110 (44.8%), respectively, compared to 100 (40.8%), (95 (38.7%), and 50 (20.4%), respectively, in the control group. The genotypic and allelic distribution of both *MTHFR* c.677C>T and *MTRR* c.66A>G polymorphisms among all CHD cases and controls are displayed in Table 2. Further analysis of genotype and allele frequencies of *MTHFR* c.677C>T and *MTRR* c.66A>G across different types of CHD revealed significant differences in both overall CHD and type-specific distribution frequencies between patients and controls, as shown in Table 3. Due to the small number of samples, pulmonary stenosis (PS) and Tetralogy of Fallot (TOF) were not analyzed individually.

**Table 2:** MTHFR c.677C>T and MTRR c.66A>G distribution in CHD and control groups. *MTHFR*: methylenetetrahydrofolate reductase; *MTRR*: methionine synthase reductase; CHD: congenital heart disease

Gene Polymorphisms	Groups	Genotype, frequency (percentage)			Allele, frequency (percentage)	
*MTHFR* c.677C>T	CHD Cases (n=245)	CC	CT	TT	C	T
		50 (20.4)	107 (43.6)	88 (35.9)	207 (42.2)	283 (57.7)
	Healthy Controls (n=245)	80 (32.6)	114 (46.5)	51 (20.8)	274 (55.9)	216 (44.1)
*MTRR* c.66A>G	CHD Cases (n=245)	AA	AG	GG	A	G
		85 (34.6)	50 (20.4)	110 (44.8)	220 (45.0)	270 (55.0)
	Healthy Controls (n=245)	100 (40.8)	95 (38.7)	50 (20.4)	295 (60.2)	195 (39.8)

**Table 3:** MTHFR c.677C>T and MTRR c.66A>G genotype frequencies in the CHD group at the type level. *: P< 0.05; **: P< 0.0 *MTHFR*: methylenetetrahydrofolate reductase; *MTRR*: methionine synthase reductase; CHD: congenital heart disease; VSD: ventricular septal defect; ASD: atrial septal defect; PDA: patent ductus arteriosus

Genes	Type of CHD	Genotype, frequency (percentage)			Allele, frequency (percentage)			OR (95% CI)					
*MTHFR* (rs1801133)		CC	CT	TT	C	T	χ^2^ value (P)	TT Vs CC	CT Vs CC		TT/CT Vs CC	TT Vs CT/CC	T Vs C
*MTHFR* c.677C>T	All CHD (n=245)	50 (20.4)	107 (43.6)	88 (35.9)	207 (42.2)	283 (57.7)	16.99^**^	2.75 (1.6-4.5)	1.50 (0.9-2.3)		1.89 (1.2-2.8)	2.13 (1.4-3.1)	1.73 (1.3-2.2)
*MTHFR* c.677C>T	VSD (n=145)	24 (16.5)	68 (46.8)	53 (36.5)	116 (40.0)	174 (60.0)	17.31**	5.67 (3.1-10.0)	3.11 (1.8-5.2)		2.40 (1.4-4.0)	2.16 (1.3-3.4)	1.90 (1.4-2.5)
*MTHFR* c.677C>T	ASD (n=82)	15 (18.2)	37 (45.1)	30 (36.5)	67 (40.8)	97 (59.1)	10.55	9.51 (4.9-18.4)	5.11 (2.7-9.4)		2.26 (1.2-4.2)	2.26 (1.3-3.9)	1.83 (1.2-2.6)
*MTHFR* c.677C>T	PDA (n=25)	9 (36.0)	6 (24.0)	10 (40.0)	24 (48.0)	26 (52.0)	6.36*	15.52 (7.1-33.7)	8.43 (4.0-17.6)		0.85 (0.3-2.0)	2.55 (1.0-6.0)	1.37 (0.7-2.4)
*MTHFR* c.677C>T	Others (n=7)	4 (57.1)	2 (28.5)	1 (14.2)	10 (71.4)	4 (28.5)	1.84	34.18 (11.8-98.9)	18.76 (6.6-53.0)		0.34 (0.1-1.6)	0.62 (0.1-5.2)	0.50 (0.1-1.6)
*MTRR* (rs1801394)		AA	AG	GG	A	G	χ^2^ value (P)	GG Vs TT	AG Vs AA	GG/AG Vs AA		GG Vs AG/AA	A Vs G
*MTRR* c.66A>G	All CHD (245)	85 (34.6)	50 (20.4)	110 (44.8)	220 (44.8)	270 (55.1)	37.68**	2.62 (1.6-4.0)	0.61 (0.3-0.9)	1.29 (0.8-1.8)		3.17 (2.1-4.7)	1.85 (1.4-2.3)
*MTRR* c.66A>G	VSD (n=145)	47 (32.4)	36 (24.8)	62 (42.7)	130 (48.8)	160 (55.1)	22.82**	4.70 (2.9-7.6)	1.12 (0.6-1.8)	1.42 (0.9-2.1)		2.86 (1.8-4.5)	1.86 (1.3-2.4)
*MTRR* c.66A>G	ASD (n=82)	26 (31.7)	16 (19.5)	40 (48.7)	68 (41.4)	96 (58.5)	26.00**	8.67 (4.9-15.0)	2.02 (1.1-3.5)	1.47 (0.8-2.5)		3.79 (2.2-6.4)	2.13 (1.4-3.1)
*MTRR* c.66A>G	PDA (n=25)	12 (48.0)	2 (8.0)	11 (44.0)	26 (52.0)	24 (48.0)	11.85*	18.54 (9.3-36.9)	4.43 (2.2-8.8)	0.74 (0.3-1.7)		3.08 (1.3-7.2)	1.39 (0.7-2.5)
*MTRR *c.66A>G	Others (n=7)	4 (57.1)	0 (0.0)	3 (42.8)	8 (57.1)	6 (42.8)	4.78	55.10 (19.2-158.1)	13.69 (4.7-39.6)	0.52 (0.1-2.3)		2.85 (0.6-13.1)	1.13 (0.3-3.3)

We evaluated the potential association between the *MTHFR* c.677C>T and *MTRR* c.66A>G genetic polymorphisms and the risk of CHD using adjusted ORs and their corresponding 95% CIs, derived from logistic regression analysis with adjustment for age. For the *MTHFR* c.677C>T polymorphism, the homozygous TT genotype demonstrated a statistically significant association with the increased risk of CHD (OR = 2.75, 95% CI: 1.6-4.5). Notably, this association was more pronounced for specific CHD subtypes, including ventricular septal defect (VSD) (OR = 5.67, 95%CI: 3.1-10.0), atrial septal defect (ASD) (OR = 9.51, 95%CI: 4.9-18.4), and patent ductus arteriosus (PDA) (OR = 15.52, 95% CI: 7.1-33.7). Additionally, the T allele exhibited a significantly higher frequency among CHD cases (n=283 (57.7%)) compared to controls (n=216 (44.1%)) (OR = 1.73, 95%CI: 1.3-2.2).

Similarly, the *MTRR* c.66A>G polymorphism was significantly associated with CHD, with individuals carrying the GG genotype displaying an increased disease risk (OR = 2.62, 95% CI: 1.6-4.0). This association was particularly strong in ASD (OR = 8.67, 95%CI: 4.9-15.0) and PDA (OR = 18.54, 95%CI: 9.3-36.9). Moreover, the G allele was more prevalent in CHD cases (n=270 (55.1%)) compared to controls (n=195 (39.7%)) (OR = 1.85, 95%CI: 1.4-2.3).

## Discussion

This case-control study was conducted in northern India to investigate the association between *MTHFR* c.677C>T and *MTRR *c.66A>G genetic polymorphisms and the risk of CHD in infants aged 0-11 months. Our findings indicate that both polymorphisms exhibit a statistically significant association with an increased risk of CHD.

It is well established that the *MTHFR* and *MTRR *genes encode critical enzymes in the folate/homocysteine metabolic pathway, which plays a pivotal role in methylation processes [5,6]. The *MTHFR *gene is located on chromosome 1p36.22, whereas the *MTRR* gene is mapped to chromosome 5p15.31. variations in these genes, particularly *MTHFR* c.677C>T and *MTRR* c.66A>G, have been implicated in multiple disorders, including neural tube defects, cardiovascular diseases, malignancies, and neurodevelopmental anomalies [6,7]. The C677T polymorphism results in an alanine-to-valine substitution, leading to reduced MTHFR enzyme activity and hyperhomocysteinemia [13]. Similarly, the A66G polymorphism in *MTRR* affects enzyme stability, thereby perturbing homocysteine metabolism [5,8].

In the present study, both *MTHFR* c.677C>T and *MTRR* c.66A>G polymorphisms demonstrated a significant association with CHD, particularly with septal defects and PDA, as indicated by adjusted odds ratios and 95% CIs [14,15].

The TT genotype and T allele of the *MTHFR* gene, along with the GG genotype and G allele of the *MTRR* gene, were associated with an increased susceptibility to CHD. These findings align with previous reports highlighting a positive correlation between *MTHFR *c.677C>T and *MTRR* c.66A>G gene polymorphisms and CHD susceptibility in paediatric populations, particularly within the Indian subcontinent [12,14,15]. Asim et al. reported a significant association between the *MTRR* c.66A>G polymorphism and CHD risk in children with Down syndrome in North India [12]. Similarly, Raina et al. [14] and Panja et al. [15] documented a significant correlation between *MTHFR* c.677C>T polymorphism and CHD risk in the same demographic. Beyond India, Pishva et al. observed a significant relationship between both *MTHFR* c.677C>T and *MTRR* c.66A>G gene polymorphisms and CHD susceptibility in the Iranian population [16], while Wang et al. demonstrated a similar association in the Chinese Han population [17]. Collectively, these studies underscore the role of genetic predisposition in CHD etiology [18-20].

In addition to genetic factors, our study examined maternal risk factors associated with CHD, revealing significant associations between advanced maternal age (≥ 30 years) and passive smoking during pregnancy with increased CHD risk. These findings highlight the contribution of environmental and lifestyle factors to CHD pathogenesis. Advanced maternal age has been linked to an increased prevalence of genetic mutations and chromosomal abnormalities, which are established contributors to congenital anomalies, including CHD. Existing literature suggests that older maternal age is linked to a higher prevalence of metabolic changes, gestational complications, and environmental exposures, all of which may have adverse effects on fetal development and increase the risk of structural heart defects [15]. Additionally, passive smoking during pregnancy has been implicated in fetal hypoxia and oxidative stress, which can disrupt normal cardiac morphogenesis. The significant associations observed in our study further support the multifactorial nature of CHD, emphasizing the interplay between genetic predispositions and environmental influences in disease susceptibility.

Strength

Our study provides important insights into the genetic and environmental factors contributing to CHD risk in the North Indian population. By adjusting for age, the analysis offers a more accurate assessment of the association between *MTHFR* c.677C>T and *MTRR *c.66A>G polymorphisms and CHD susceptibility.

Limitations

The sample size for certain CHD subtypes, such as pulmonary stenosis (PS) and Tetralogy of Fallot (TOF), was relatively small, thereby limiting the statistical power to perform subtype-specific analyses. Moreover, important variables such as dietary folate intake and maternal genetic background were not assessed, which could be incorporated in future investigations to provide a more comprehensive understanding of gene-environment interactions underlying CHD pathogenesis. Additionally, as this was a single-center study, a multicenter design in future research would enable inclusion of a more diverse genetic pool and a larger sample size, thereby enhancing the generalizability of the findings.

## Conclusions

The study highlights significant associations between the *MTHFR* c.677C>T and *MTRR* c.66A>G gene polymorphisms and CHD, suggesting these genetic variants may contribute to CHD susceptibility. Additionally, advanced maternal age and passive smoking are identified as critical non-genetic risk factors, emphasizing the multifactorial nature of CHD etiology. Awareness of maternal risk factors, combined with an understanding of the interplay between genetic and environmental factors, can guide public health interventions, including education, lifestyle modifications, targeted prenatal counselling, and preventive strategies for at-risk populations.
